# Novel electrochemical and electrochemiluminescence dual-modality sensing platform for sensitive determination of antimicrobial peptides based on probe encapsulated liposome and nanochannel array electrode

**DOI:** 10.3389/fnut.2022.962736

**Published:** 2022-08-15

**Authors:** Xuan Luo, Tongtong Zhang, Hongliang Tang, Jiyang Liu

**Affiliations:** ^1^Department of Chemistry, Key Laboratory of Surface and Interface Science of Polymer Materials of Zhejiang Province, Zhejiang Sci-Tech University, Hangzhou, China; ^2^Department of Hepatobiliary and Pancreatic Surgery, The Center for Integrated Oncology and Precision Medicine, Affiliated Hangzhou First People's Hospital, Zhejiang University School of Medicine, Hangzhou, China; ^3^Affiliated Fangchenggang Hospital, Guangxi University of Chinese Medicine, Fangchenggang, China

**Keywords:** electrochemical and electrochemiluminescence detection, dual-modality sensor, antimicrobial peptide, liposome, nanochannel array, food analysis

## Abstract

With the increasing application of antimicrobial peptides (AMPs) to replace antibiotics in medicine, food and agriculture, it is highly desired to develop a fast, reliable, and convenient strategy for sensitive detection of AMPs. Herein, a novel electrochemical (EC) and electrochemiluminescence (ECL) dual-modality sensing platform was developed based on probe encapsulated liposomes and nanochannel array modified electrodes, which enables sensitive determination of nisin in food samples. The bifunctional probe with both EC and ECL signals, tris(2,2-bipyridyl) dichlororuthenium (II) (Ru(bpy)_3_^2+^), was chosen to be easily encapsulated in liposomes (Ru(bpy)_3_^2+^@liposome). Based on the unique sterilization mechanism that AMPs can disrupt cell membranes, Ru(bpy)_3_^2+^@liposome can be destroyed by nisin and release a large number of Ru(bpy)_3_^2+^ probes. Vertically-ordered mesoporous silica-nanochannel film (VMSF) modified ITO electrodes (VMSF/ITO) prepared by electrochemically assisted self-assembly (EASA) method were applied as the sensing electrode. Due to the efficient enrichment of Ru(bpy)_3_^2+^ by the negatively charged nanochannel arrays, VMSF/ITO enables detection of the EC/ECL signals of the released Ru(bpy)_3_^2+^ probes with ultrahigh sensitivity. In consequence, sensitive dual-modality detection of nisin was achieved by the combination of Ru(bpy)_3_^2+^@liposome and VMSF/ITO. The developed sensing system can realize sensitive determination of nisin in ECL mode in the concentration range of 10 ng/ml to 50 μg/ml with a limit of detection (LOD) of 9.3 ng/ml, or in EC mode from 800 ng/ml to 100 μg/ml with a LOD of 70 ng/ml. Combined with the excellent anti-fouling and anti-interference performance of VMSF, rapid and sensitive detection of nisin in milk or egg white was also achieved by the sensor.

## Introduction

With the long-term and widespread application of antibiotics, the resistance of pathogenic bacteria is becoming more and more serious, which poses serious hazards to food and environmental safety as well as human health. Therefore, the development of new harmless antibiotics is of great significance. The discovery of antimicrobial peptides (AMPs, also known as bactericidal peptides) provides new perspectives and broad sources for the development of new antibiotics. AMPs are small molecular polypeptides with biological activity induced in organisms. They have a wide range of antibacterial activities and also have inhibitory effects on fungi, protists, tumor cells and viruses (1). Unlike antibiotics that act on glycoproteins on the cell membrane surface (2), AMPs interact with the phospholipid bilayer of the cell membrane, killing cells or bacteria by causing rupture of the cell membrane and leakage of cellular contents (3, 4). At present, more than 2,400 AMPs have been isolated from organisms (such as insects, birds, fish, amphibians, and mammals, etc.), some of which have already been prepared on a large scale by genetic engineering (5–7). Up to now, AMPs have been widely used in medicine, food, animal husbandry and other fields (8–10). For example, nisin is a positively charged amphiphilic antimicrobial peptide containing 34 amino acid residues (11), which can effectively inhibit most Gram-positive bacteria and some Gram-positive bacteria that cause food spoilage. Since nisin is extremely sensitive to proteolytic enzymes, it can be quickly enzymatically decomposed into amino acids after consumption, and has no toxic and side effects on the human body. As a safe and efficient food preservative, nisin has replaced or partially replaced chemical preservatives in more than 60 countries and regions around the world for the preservation of dairy products, canned foods, meat products, baked goods and beverages.

The detection of AMPs is the key to their preparation and application. For example, nisin is mainly produced by microbial fermentation. The fermentation endpoint is affected by the amount and activity of bacteria, while nisin is unstable in the fermentation broth. Thus, it is necessary to adjust parameters or end the fermentation process in time to ensure the maximum yield and the minimum cost. At present, the purity of nisin produced by different companies in the market varies greatly and even some products are far below the national standard. In addition, when nisin is used as a food additive, the concentration and activity of nisin will also decrease during food processing and storage due to the influence of temperature, pH value and composition of different foods. Accurate quantification analysis of nisin is essential for determining its level in food and assessing its stability over the shelf life of food (12, 13). Until now, the detection strategies of nisin include antibacterial detection (e.g., agar diffusion method and turbidity colorimetric), immunoassay (e.g., enzyme-linked immunosorbent assay-ELISA, immunoblotting), bioluminescence detection (e.g., aptamer-based bioluminescence assay, biosensors), and high performance liquid chromatography (HPLC) (14–19). However, these methods usually suffer from complex operations, long detection times, and high manipulation skills. Therefore, a fast, reliable, and convenient strategy for the sensitive detection of nisin is highly desired.

Electrochemistry (EC) and electrochemiluminescence (ECL) are the most widely used analytical methods due to their advantages of high sensitivity, simple operation, fast response, and potential for online monitoring (20–25). In comparison, the instrument of EC is simpler, while the sensitivity of ECL is generally higher. EC/ECL dual-modality detection can further improve the reliability and accuracy. However, nisin itself has no electroactive or ECL properties. Until now, the detection of nisin by EC or ECL methods has not been reported. Very recently, the construction of specific nanostructures on the electrode surface provides a new strategy to achieve indirect detection of non-electroactive substances (26–30). Amongst, vertically-ordered mesoporous silica-nanochannel film (VMSF) modified electrodes have attracted extensive attention. VMSF consists of a highly ordered array of nanochannels perpendicular to the electrode substrate with uniform pore size (typically 2–3 nm), ultrathin thickness (typically 50–200 nm), and high pore density (75,000/μm^2^) (31). This unique nanochannel array exhibits permselectivity based on material size, charge, or hydrophilicity, which makes VMSF-modified electrodes resistant to contamination and interference (32). On the one hand, the size exclusion of ultra-small pores enables VMSF to prevent particles (e.g., particles or cells) or biomacromolecules (e.g., proteins, DNA, etc.) from entering the nanochannels, improving the anti-fouling performance of sensing electrodes. On the other hand, deionization of silanol groups (p*K*_a_~2) on the nanochannel surface makes VMSF negatively charged under normal pH conditions, thereby repelling negatively charged electroactive small molecules. For example, interference caused by common redox species in complex samples such as ascorbic acid (AA) or uric acid (UA) can be avoided. Furthermore, it is worth noting that VMSF nanochannels can enrich analytes through electrostatic or hydrogen bonding interactions, leading to significant signal amplification. For example, the commonly used positive ECL probe tris(2,2-bipyridyl) dichlororuthenium (II) (Ru(bpy)_3_^2+^) can be efficiently enriched by VMSF, increasing the detection sensitivity by two orders of magnitude (33). Therefore, VMSF modified electrodes have great potential for direct and highly sensitive EC/ECL dual-modality detection of complex samples without separation or enrichment pre-treatment.

In this paper, an EC/ECL dual-modality sensing platform for the high-sensitivity determination of antimicrobial peptides was established for the first time by combining the interaction mechanism of antimicrobial peptides with cell membranes and the excellent performance of VMSF-modified electrodes such as signal amplification, anti-fouling and anti-interference. As a proof-of-concept demonstration, liposomes were used to mimic cell membranes and encapsulated a large amount of Ru(bpy)_3_^2+^ as signal molecules for both EC and ECL detection (Ru(bpy)_3_^2+^@liposome). When nisin interacted with the liposomes to cause their rupture, the inner Ru(bpy)_3_^2+^ probes were released and could be sensitively detected in an EC/ECL mode (signal-on) through the efficient enrichment of these positive probes by the VMSF nanochannel arrays. By measuring the EC or ECL signals of Ru(bpy)_3_^2+^ on the VMSF modified electrode, a highly sensitive and signal-on dual-modality detection of nisin was obtained. Combined with the anti-interference and anti-fouling properties of VMSF, rapid and highly sensitive detection of nisin in milk or egg white was achieved.

## Materials and methods

### Chemicals and materials

Cetyltrimethylammonium bromide (CTAB), tetraethoxysilane (TEOS), CHCl_3_, CH_3_OH, NaH_2_PO_4_, Na_2_HPO_4_, potassium hydrogen phthalate (KHP), potassium ferricyanide (K_3_[Fe(CN)_6_]), ferrocenemethanol (FcMeOH), cysteine (L-Cys), glycine (Gly), bovine serum albumin (BSA), glucose (Glu), ascorbic acid (AA) and lactose were purchased from Aladdin Chemistry (China). Cholesterol, 1,2-dimyristoyl-sn-glycero-3-phosphocholine (DMPC), 1,2-ditetradecanoyl-sn-glycero-3-[phospho-rac-(1-glycerol)] sodium salt (DMPG), nisin, potassium ferricyanide (K_3_[Fe(CN)_6_]), potassium ferrocyanide (K_4_[Fe(CN)_6_]) and potassium chloride (KCl) were obtained from Macklin (China). Hexaammineruthenium (III) chloride (Ru(NH_3_)_6_Cl_3_) and Tris(2,2-bipyridine)dichlororuthenium(II) hexahydrate (Ru(bpy)_3_Cl_2_·6H_2_O) were purchased from Sigma–Aldrich (USA). Milk and egg white were obtained from the local supermarket (Hangzhou, China). Ultrapure water (18.2 MΩ cm) was used throughout the work to prepare the aqueous solutions. ITO electrodes (ITO coated glasses, <17 Ω/square, thickness: 100 ± 20 nm) were obtained from Zhuhai Kaivo Optoelectronic Technology (China). Before use, The ITO electrodes were firstly cleaned using NaOH solution (1 M) and then sonicated in acetone, ethanol, and ultrapure water, respectively.

### Measurements and instrumentations

Transmission electron microscopy (TEM) photographs were taken on a JEM-2100 transmission electron microscope (JEOL Co., Ltd., Japan) at a working voltage of 200 kV. Scanning electron microscopy (SEM) was performed on a field emission scanning electron microscope (S-4800, Hitachi, Japan). Ultraviolet-Vis (UV-Vis) absorption spectra were recorded on a UV-Vis spectrometer (UV-2450; Shimadzu, Japan). ECL measurements were performed using a CHI 660D electrochemical workstation (CH Instrument, China) and an MPI multifunctional ECL analyzer (Xi'an Remex Analytical Instrument Ltd., China). All electrochemical measurements were performed on an Autolab PGSTAT302N electrochemical workstation (Metrohm, Switzerland). Amongst, cyclic voltammetry (CV) scanning was performed over a potential range of 0–1.4 V at a scan rate of 100 mV/s. The DPV curves were obtained using a certain step (0.005 V), modulation amplitude (0.05 V), modulation time (0.05 s), and interval time (0.2 s). The three-electrode system was used for CV and DPV experiments. VMSF modified ITO (VMSF/ITO) was used as the working electrode, an Ag/AgCl (saturated with KCl solution) was employed as the reference electrode, and a platinum wire was applied as the counter electrode.

### Preparation of VMSF modified ITO electrodes

As previously reported (34), VMSF modified ITO electrodes were rapidly fabricated by an electrochemically assisted self-assembly (EASA) method. Briefly, 20 ml NaNO_3_ (0.1 M, pH = 3) and 20 ml ethanol were mixed. Then, TEOS (2.833 g) and CTAB (1.585 g) were added and the mixture was stirred for 2.5 h to obtain the precursor solution. For VMSF growth, a constant current (-0.7 mA/cm^2^) was applied on an ITO electrode for 10 s. Then the electrode was quickly rinsed with ultrapure water. After being dried under N_2_, the obtained electrode was aged at 120°C for 12 h. The as-prepared electrode retained surfactant micelles that blocked the nanochannels (SM@VMSF/ITO). The inner SM was removed through immersing the electrode in a HCl-ethanol solution (0.1 M) with stirring for 5 min. The obtained electrode with open nanochannels was termed as VMSF/ITO.

### Preparation of Ru(bpy)_3_^2+^-encapsulated liposomes

Ru(bpy)_3_^2+^-encapsulated liposomes (Ru(bpy)_3_^2+^@liposome) were prepared according to the literatureswith slight modifications (35, 36). Briefly, DMPG (2 mg), DMPC (8 mg) and cholesterol (8 mg) were co-dissolved in the mixture of chloroform and methanol (*V*/*V* = 1:1, 300 μl) through sonication for 5 min. After evaporation under nitrogen, the solution was put in vacuum for 4 h to remove residue organic solvent. The obtained lipid film was dissolved in PBS (1 ml, 0.01 M, pH 7.4) containing Ru(bpy)_3_^2+^ (20 mM) and incubated at 30°C for 3 h. Then, the obtained mixture was sonicated in an ice water bath for 1 h to obtain a homogeneous orange suspension. The suspension was centrifuged at 15,000 rpm for 30 min to remove the free Ru(bpy)_3_^2+^. The obtained precipitate was thoroughly washed using PBS (0.01 M, pH 7.4) until the supernatant was colorless. The Ru(bpy)_3_^2+^@liposome was finally redispersed in 1 ml of PBS (0.01 M, pH 7.4) and stored at 4°C.

### EC and ECL determination of nisin

Seven milliliter of PBS (0.01 M, pH 7.4) containing 10 μl of above Ru(bpy)_3_^2+^@liposome solution or 10 μl Ru(bpy)_3_^2+^@liposome and 3 mM TPrA was used as the EC or ECL detection medium. Before each measurement, different concentrations of nisin were added under slight stirring for 5 min to release Ru(bpy)_3_^2+^ from liposomes. Then, the DPV or ECL signal was measured using VMSF/ITO electrode. For real sample analysis, milk and egg white were centrifuged at 10,000 rpm for 10 min to remove the interfering substances such as fat and protein (37). Due to the excellent anti-fouling and anti-interference performance of VMSF, no additional filtering step was required. Subsequently, the samples were diluted by a factor of 50 with PBS (0.01 M, pH = 7.4) and spiked with different concentrations of nisin to provide three final concentrations. Finally, nisin in milk was determined using EC method and the egg white was detected with ECL strategy.

## Results and discussion

### Preparation and characterizations of VMSF modified ITO electrodes

**Figure 1:**
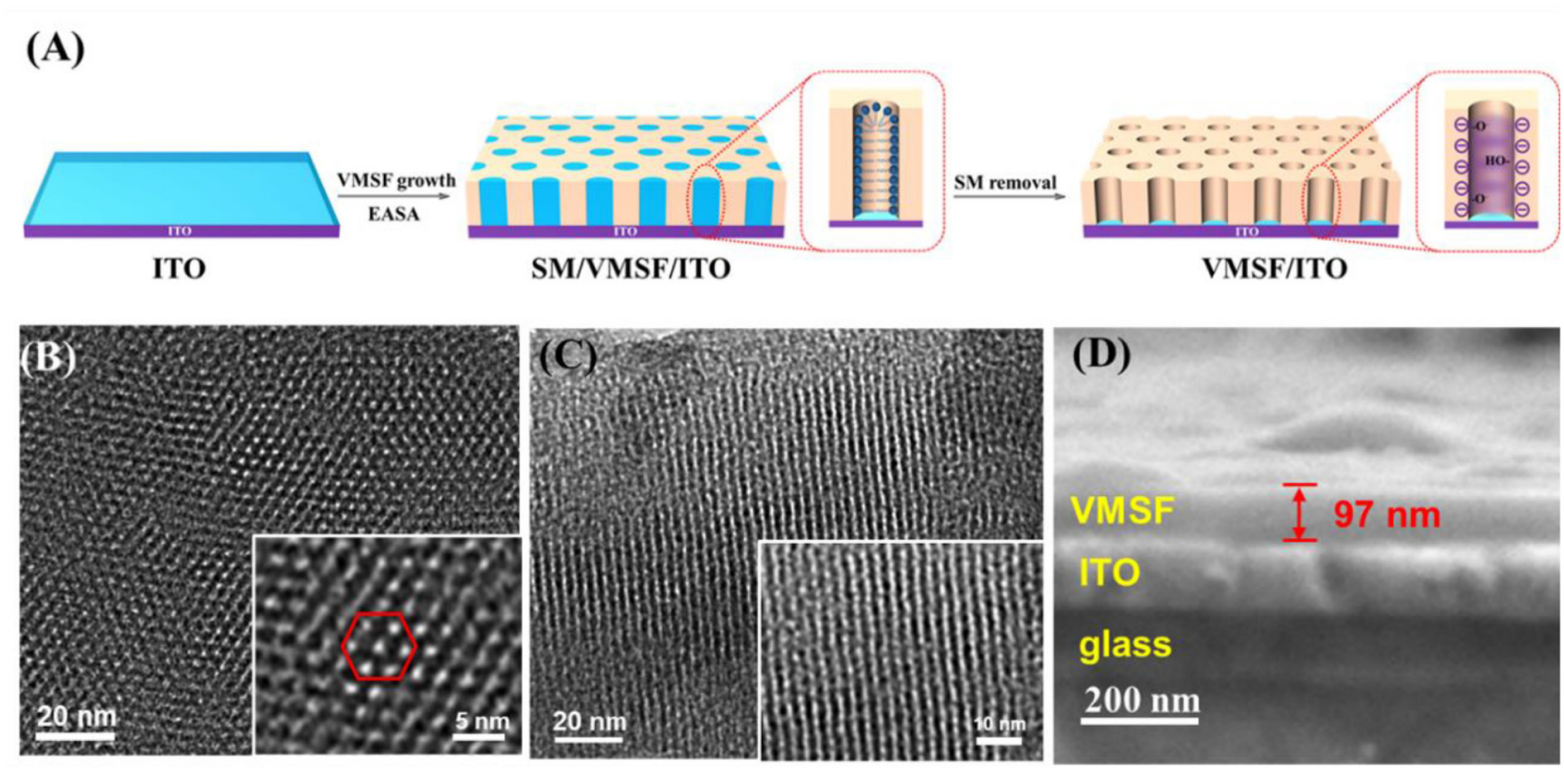
**(A)** Schematic illustration for the fabrication of VMSF/ITO electrode. Top-view **(B)** and cross-sectional **(C)** images of VMSF. The inset is the corresponding high-resolution TEM image. **(D)** SEM image of cross-section of VMSF.

At present, the synthesis methods of VMSF mainly include Stöber solution growth method, two-phase layered growth method, electrochemical assisted self-assembly (EASA) method, etc. Due to the simple operation and fast growth (within a few seconds), the EASA method was used to grow VMSF on the surface of ITO electrode with hexadecyl ammonium bromide micelles (CTAB) as templates (Figure 1A). When a cathodic potential was applied to the ITO electrode, the electrolysis of water resulted in a pH gradient at the electrode surface, which promoted the condensation of the silica precursor. After VMSF growth, surfactant micelles (SMs) were packed inside the nanochannels. After removing the SMs, open nanochannels were obtained and the electrode was termed as VMSF/ITO. The structure and morphology of VMSF were characterized by transmission electron microscopy (TEM) and scanning electron microscopy (SEM). The top-view TEM image of VMSF reveals a uniformly distributed nanochannel array without cracks and defects within the observed range (Figure 1B). The diameter of the nanopore is 2–3 nm (inset in Figure 1B). As displayed in the cross-sectional TEM image, nanochannels are observed to be parallel to each other and perpendicular to the electrode substrate (Figure 1C). The SEM image shows that VMSF/ITO has an obvious three-layer structure, corresponding to glass layer, ITO layer, and VMSF layer, respectively (Figure 1D). The thickness of VMSF is ~97 nm.

**Figure 2:**
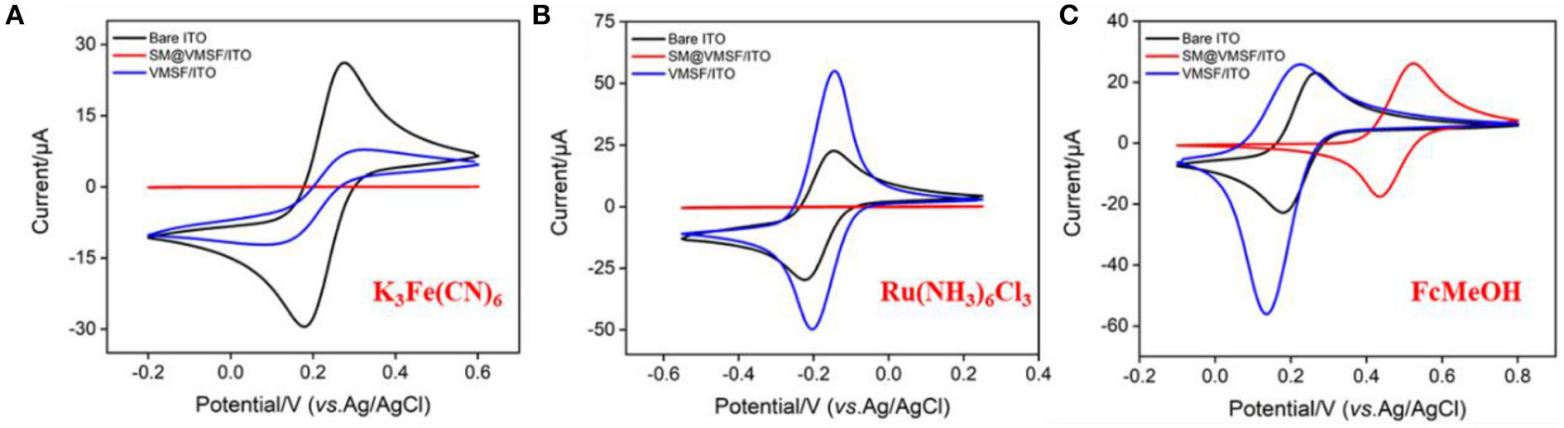
Cyclic voltametric curves obtained on ITO, SM@VMSF/ITO or VMSF/ITO electrode in K_3_Fe(CN)_6_ **(A)**, Ru(NH_3_)_6_Cl_3_ **(B)**, and FcMeOH **(C)**. The redox probe solution (0.5 mM) is prepared in KHP (0.05 M, pH = 7.4). The scan rate is 50 mV/s.

The integrity and permeability of VMSF were investigated by electrochemical characterization. CV responses of three standard electrochemical probes including an anion probe K_3_Fe(CN)_6_, a cation probe Ru(NH_3_)_6_Cl_3_ and a neutral organic probe ferrocenemethanol (FcMeOH) on different electrodes were studied (Figure 2). As shown, the SM@VMSF/ITO electrode shows no signal for both K_3_Fe(CN)_6_ and Ru(NH_3_)_6_Cl_3_ because the hydrophobic micelles block the nanochannels and prevent both the anionic and cationic probes from entering the pores and reaching the electrode surface. In contrast, the neutral probe FcMeOH can dissolve in micelles and reach the electrode surface through the enrichment of micelles to generate redox signals. These results prove that the VMSF completely covers the surface of the ITO electrode without defects or cracks. After SMs removal, VMSF/ITO electrode exhibits remarkable current signals in all three probes, demonstrating the open of the nanochannel array. In comparison with bare ITO, VMSF/ITO exhibits suppressed peak current in K_3_Fe(CN)_6_ solution while enhanced signal in case of Ru(NH_3_)_6_Cl_3_, demonstrating the charge-selective permeability of the nanochannels. The deprotonation of partial silanol groups (p*K*_a_~2) on the nanochannel surface makes the nanochannels negatively charged, thus repelling the negatively charged probes and attracting the positively charged probes. In case of FcMeOH, the reduction peak current is greater than the oxidation peak current because the oxidation products of FcMeOH are positively charged and can be enriched by the nanochannels. This remarkable charge-selective permeability is beneficial to improve the detection sensitivity of positively charged analytes, and to exclude co-existing negatively charged molecules, thereby improving the anti-interference performance of the electrode.

### Characterization of Ru(bpy)_3_^2+^-encapsulated liposomes

**Figure 3:**
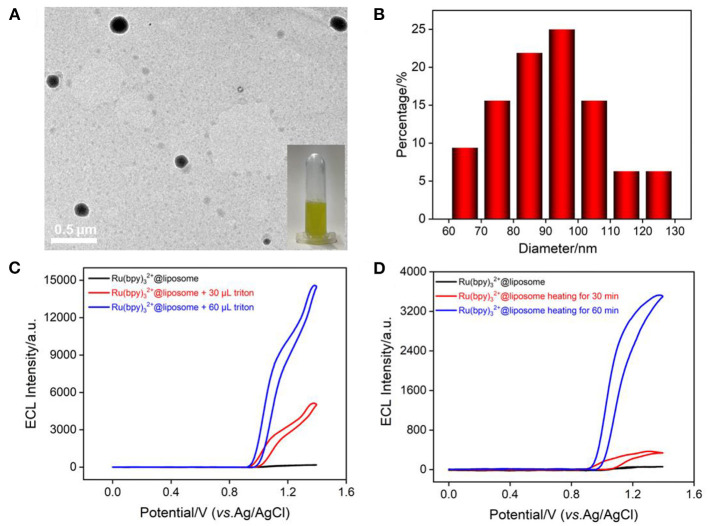
**(A)** TEM image of Ru(bpy)_3_^2+^@liposome. The inset is a digital photo of Ru(bpy)_3_^2+^@liposome solution. **(B)** Size distribution of Ru(bpy)_3_^2+^@liposome. **(C,D)** ECL intensity-potential curves of Ru(bpy)_3_^2+^@liposome when adding different concentrations of triton or heating (50°C) for different times. The voltage of the photomultiplier tube (PMT) is 650 V.

Liposome is closed vesicles with a phospholipid bilayer similar to cell membranes and can serve as a typical artificial biofilm model. In this work, liposome was used to mimic the cell membrane and the most common ECL probe with high luminescence efficiency, Ru(bpy)_3_^2+^, was encapsulated in liposome (Ru(bpy)_3_^2+^@liposome). As shown in the inset of Figure 3A, Ru(bpy)_3_^2+^@liposome solution is an orange dispersion. TEM image shows that the liposome has a spherical structure (Figure 3A) with a size distribution ranging from 60 to 130 nm, with an average size of ~97 nm (Figure 3B). The ECL signal of Ru(bpy)_3_^2+^ was used to characterize its encapsulation in liposomes and the structural changes of liposomes. As shown in Figures 3C,D, the ECL signal of Ru(bpy)_3_^2+^ could not be detected in the solution of Ru(bpy)_3_^2+^@liposome, indicating that Ru(bpy)_3_^2+^ probe molecules were successfully encapsulated inside the hydrophilic cavity of liposome and thus unable to reach the electrode surface. As known, the addition of surfactants or heat can disrupt the structure of liposomes. As shown in Figure 3C, after the addition of surfactant triton into the Ru(bpy)_3_^2+^@liposome solution, the ECL signal of Ru(bpy)_3_^2+^ increased significantly with the increase of triton concentration. The ECL signal also gradually increased with the heating time. Thus, disruption of the liposome bilayer by addition of surfactant or heating results in the release of Ru(bpy)_3_^2+^, thereby increasing the ECL signal.

**Figure 4:**
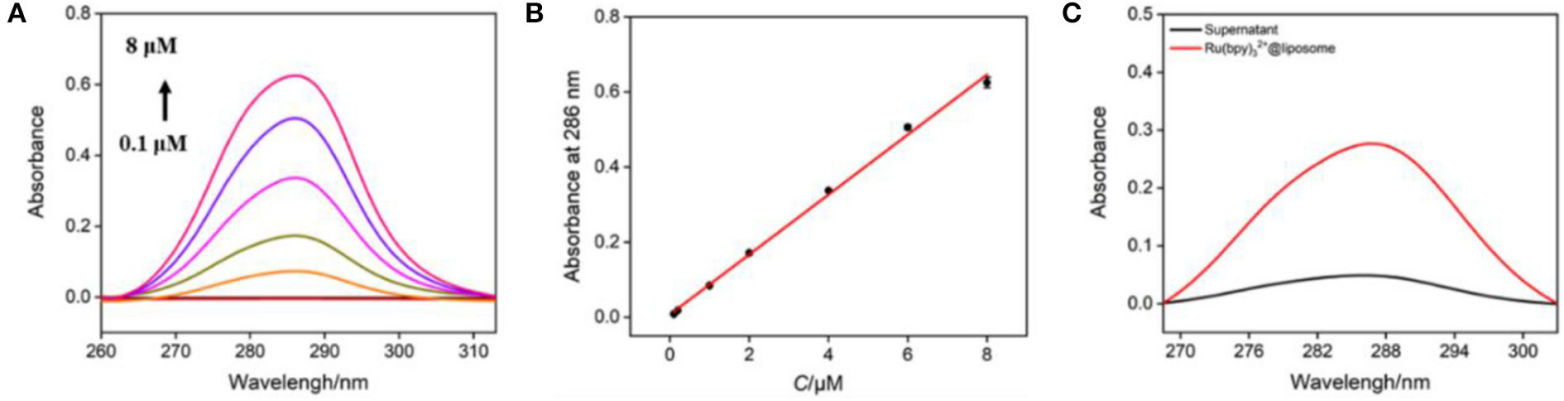
**(A)** UV absorption spectra of different concentrations of Ru(bpy)_3_^2+^ (from bottom to up, 0.1 μM, 0.2 μM, 1 μM, 2 μM, 4 μM, 6 μM, 8 μM). **(B)** The linear regression curve of absorbance vs. the concentration of Ru(bpy)_3_^2+^. **(C)** UV absorption spectra of Ru(bpy)_3_^2+^@liposome (diluted with PBS for 200 times) and the supernatant (diluted with PBS for 40 times) obtained after centrifugation.

The bipyridine structure endows Ru(bpy)_3_^2+^ with characteristic UV absorption (Figure 4A) and its absorbance (A) is linear with the concentration (*C*) (Figure 4B, *A* = 0.0798 *C* + 0.0072 (*R*^2^ = 0.997). As shown in Figure 4C, the Ru(bpy)_3_^2+^@liposome solution exhibits the characteristic absorption of Ru(bpy)_3_^2+^. On the contrary, there is basically no absorption in the supernatant after separation of Ru(bpy)_3_^2+^@liposome through centrifugation, proving once again that Ru(bpy)_3_^2+^ was encapsulated inside the hydrophilic cavity of liposomes. The amount of Ru(bpy)_3_^2+^ encapsulated in liposomes was calculated to be 0.036 mg Ru(bpy)_3_^2+^/mg liposome.

The electrochemical impedance spectroscopy (EIS) of Ru(bpy)_3_^2+^@liposome has been investigated using ITO as the supporting electrode (Supplementary Figure S1 in supporting information-SI). The schematic illustration of the equivalent circuit is also displayed as inset of Supplementary Figure S1, which includes solution resistance (*R*_s_), double-layer capacitance (*C*_dl_), Warburg impedance (*Z*_w_), and apparent charge transfer resistance (*R*_ct)_. For comparison, the EIS plot of ITO is also studied for comparison. As shown, each curve consists of a semicircle in the high frequency region representing the electron transfer process and a linear part in the low frequency region relating to the diffusion process. In addition, the equivalent diameter of the semicircle in the high frequency region is equal to *R*_ct_. After ITO electrode was modified with Ru(bpy)_3_^2+^@liposome, the semicircle in the EIS plot increases, indicating the increase of *R*_ct_ because liposome acts as an inert layer and hinders electron transfer. This phenomenon suggests the successful preparation of Ru(bpy)_3_^2+^@liposome.

### ECL of Ru(bpy)_3_^2+^@liposome in presence of nisin

**Figure 5:**
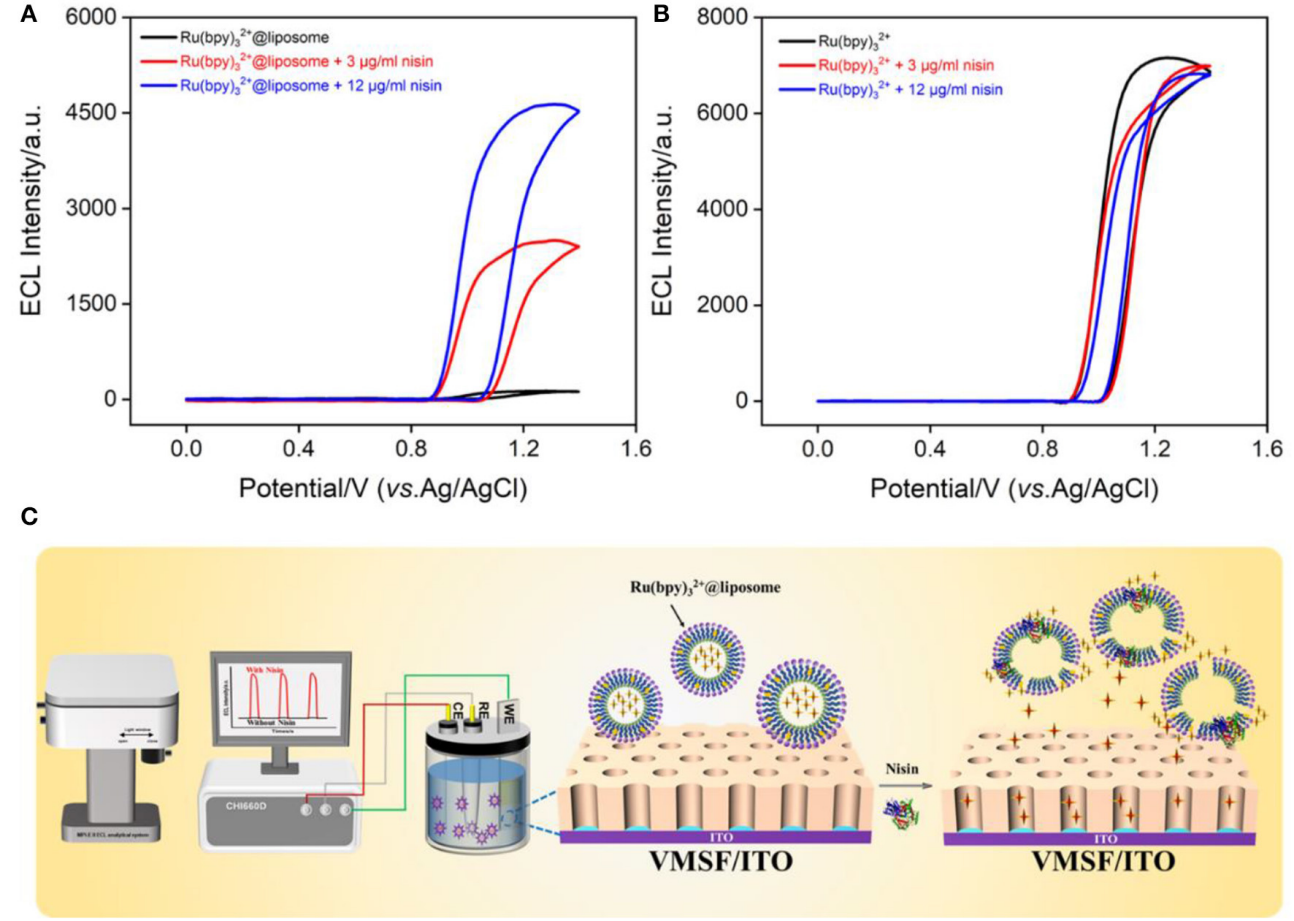
ECL signal-potential curves obtained before and after adding different concentrations of nisin to Ru(bpy)_3_^2+^@liposome **(A)** or Ru(bpy)_3_^2+^ **(B)**. The PMT voltage is 650 V **(A)** or 400 V **(B)**, respectively. **(C)** The illustration of ECL determination of nisin based on the release of Ru(bpy)_3_^2+^ resulting from the interaction between nisin and liposome.

In comparison with other luminophore technology (e.g., fluorescence) (38–46), ECL has advantages of no background, high potential and spatial controllability, and wide response range. Figure 5A shows the ECL curves obtained on the VMSF/ITO electrode in Ru(bpy)_3_^2+^@liposome in the absence or presence of nisin. As seen, the ECL signal of Ru(bpy)_3_^2+^ appears after the addition of nisin. And the increase of nisin concentration leads to the enhancement of the ECL signal. However, no significant change in ECL signal was observed in the control Ru(bpy)_3_^2+^ solution after addition of nisin (Figure 5B). Thus, nisin could not act as a co-reactant to promote the ECL intensity of Ru(bpy)_3_^2+^. The signal enhancement in Ru(bpy)_3_^2+^@liposome solution after the addition of nisin is due to the disruption of the liposome structure, resulting in the leakage of Ru(bpy)_3_^2+^. When Ru(bpy)_3_^2+^ (~1.2 nm) is released, it could be enriched by the negatively charged nanochannel array (2–3 nm in diameter) because of the electrostatic interaction between VMSF and Ru(bpy)_3_^2+^ (47– 51). Thus, the released Ru(bpy)_3_^2+^ could get to the ITO surface, leading to significant ECL signal.

As known, nisin itself does not have electrochemical or electrochemiluminescence properties, so in this work, Ru(bpy)_3_^2+^@liposome was synthesized and applied as a sensing membrane and probe for the detection of nisin. As shown in Figure 5C, when nisin was added, liposomes were disrupted and the released Ru(bpy)_3_^2+^ could act as the source of the ECL signal. The mechanism by which nisin destroys liposomes could be attributed to two aspects. On the one hand, the positively charged C-terminus of nisin binds to the negatively charged liposomes through electrostatic interaction, resulting in a local disturbance of the phospholipid group in the head, which further forms a wedge shape channel. On the other hand, the N-terminus of nisin is rich in hydrophilic amino acid residues, while the C-terminus is rich in hydrophobic amino acid residues. When the hydrophilic group interacts with the phospholipid head group, the hydrophobic side chain is immersed in the hydrophobic core of the liposome to form pores (4). Thus, the presence of nisin can disrupt the liposomes, allowing the probe Ru(bpy)_3_^2+^ to leak from the inner hydrophobic cavity. The negatively charged nanochannels on VMSF/ITO electrode can efficiently enrich Ru(bpy)_3_^2+^ through electrostatic interactions, resulting in sensitive ECL signals.

### ECL determination of nisin using VMSF/ITO electrode

**Figure 6:**
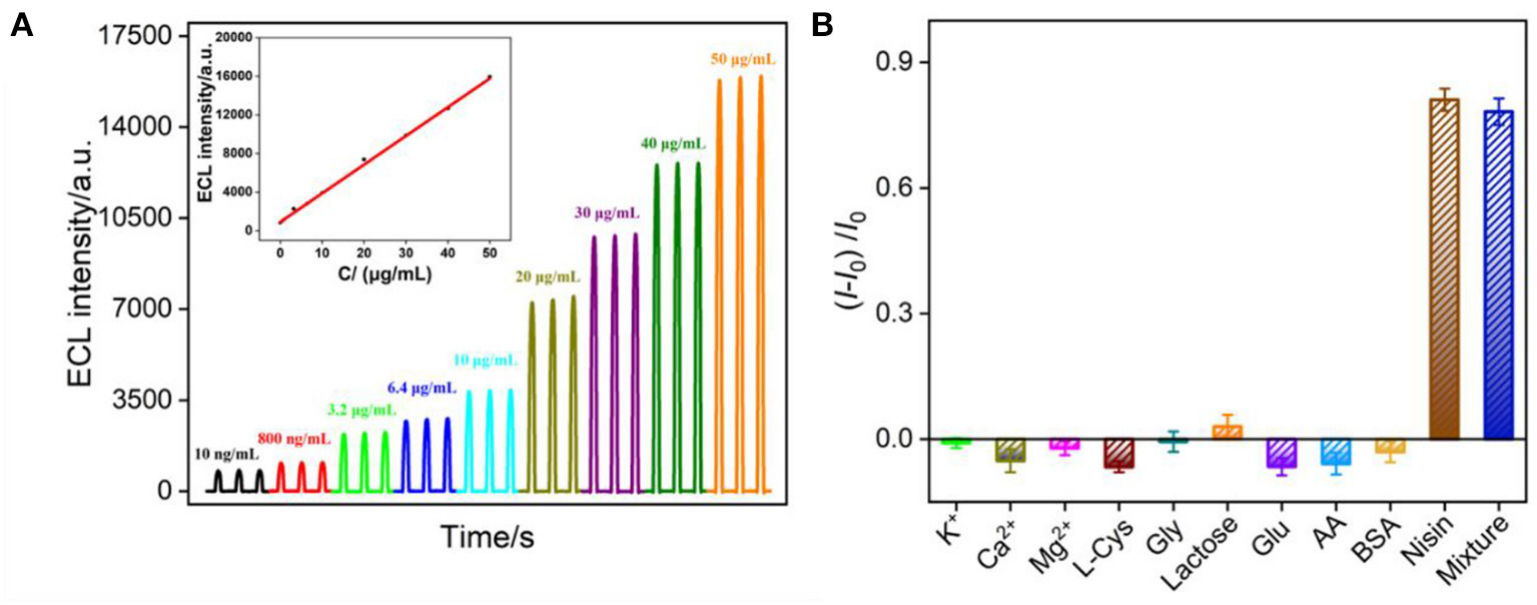
**(A)** ECL response of VMSF/ITO electrode obtained using Ru(bpy)_3_^2+^@liposome probe in presence of different concentrations of nisin. Inset is the calibration curve for the detection of nisin. The voltage of PMT is 650 V. **(B)** The increased ECL intensity ratio obtained using Ru(bpy)_3_^2+^@liposome probe in presence of different substances. The concentration of K^+^, Ca^2+^, Mg^2+^, L-Cys and Gly lactose, Glu, and AA is 40 μg/ml, BSA is 1%. The concentration of nisin is 10 μg/ml. *I* and *I*_0_ represent the ECL signal in absence or presence of one or mixed substance, respectively.

The ECL detection of nisin using VMSF/ITO electrode was investigated based on the mechanism that nisin could destroy the liposome structure, resulting in the release of Ru(bpy)_3_^2+^ to generate ECL signals. Figure 6A shows the ECL curves obtained in Ru(bpy)_3_^2+^@liposome solution with different concentrations of nisin. It can be found that the ECL signal of the solution increased with the increase of nisin concentration. When the nisin concentration ranged from 10 ng/ml to 50 μg/ml, the ECL intensity (*I*) had a good linear fitting relationship with the concentration of nisin (*C*_nisin_) (*I* = 297.5 *C*_nisin_ + 896.2, *R*^2^ = 0.998, inset in Figure 6A). The limit of detection (LOD) based on a three signal-to-noise ratio (S/N = 3) is 9.3 ng/ml.

Detection selectivity is important for the application of the developed sensor. The selectivity of VMSF/ITO electrode to detect nisin was investigated by evaluating the effects of common ions (K^+^, Ca^2+^ and Mg^2+^), some amino acids (L-cysteine, L-Cys; glycine, Gly), sugars (lactose, glucose-Glu), redox small molecules (ascorbic acid-AA), protein (bovine serum albumin-BSA), or their mixture on the detection of nisin. As shown in Figure 6B, when the concentration of the interfering substance was much higher than that of nisin, the change ratio of ECL signal calculated using ECL intensity before (*I*_0_) and after (*I*) adding a single of interfering substance was significantly low, proving that the electrode had good selectivity.

### EC determination of nisin using VMSF/ITO electrode

**Figure 7:**
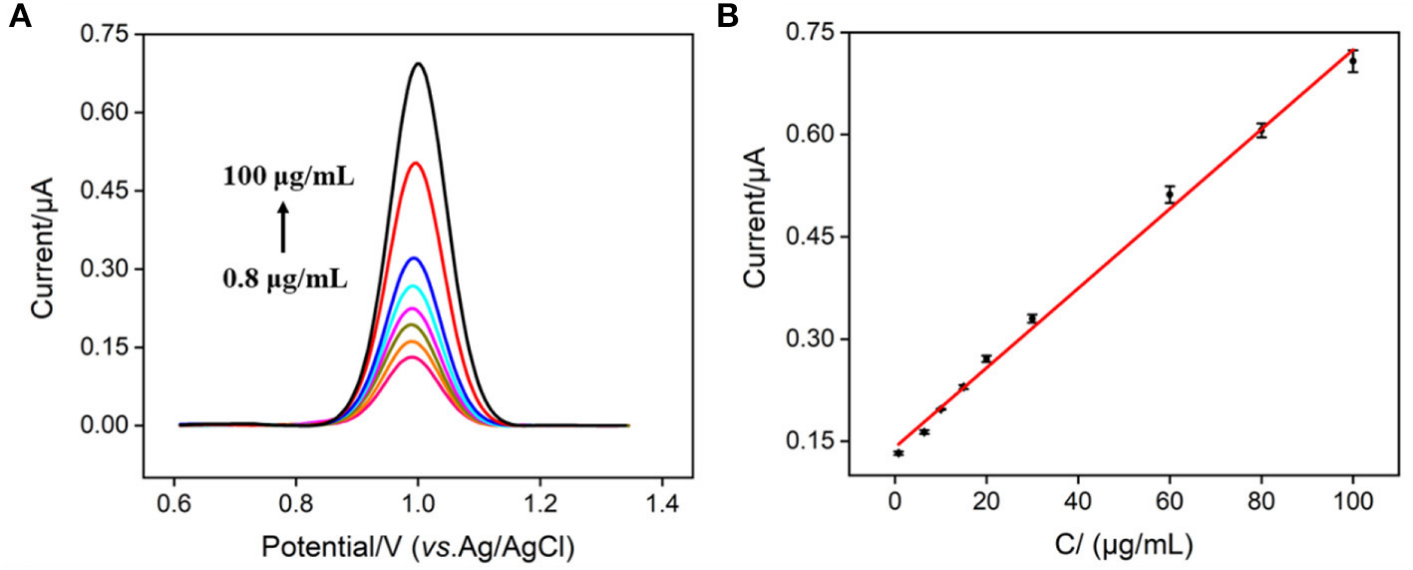
**(A)** DPV curves obtained on VMSF/ITO electrode in Ru(bpy)_3_^2+^@liposome probe containing different concentrations of nisin. **(B)** The calibration curve for the detection of nisin. The error bar represents the standard deviation of three measurements.

ECL generally has higher sensitivity, while EC detection requires simpler instrumentation. Though Ru(bpy)_3_^2+^ can be used as an ECL probe with excellent properties, its redox properties also make it an EC probe. Therefore, the EC detection of nisin can be simultaneously realized by measuring the electrochemical signal of Ru(bpy)_3_^2+^. Figure 7A shows the DPV curves obtained in Ru(bpy)_3_^2+^@liposome solution in the presence of different concentrations of nisin. The oxidation peak current of Ru(bpy)_3_^2+^ increased with the increase of the concentration of nisin. When the concentration of nisin was between 800 ng/ml and 100 μg/ml, the oxidation peak current (*I*) had a good linear relationship with the concentration of nisin (C_nisin_) (*I* = 0.0059 *C*_nisin_ + 0.1411, *R*^2^ = 0.996, Figure 7B). The LOD is 70 ng/ml (S/N = 3). Thus, the EC/ECL dual-modality sensing platform for the highly sensitive determination of nisin is established for the first time. Comparison between determination of nisin using different strategies is demonstrated in Supplementary Table S1 (SI) (14, 17, 52–54). The LOD obtained by EC detection is lower than that obtained from micellar electrokinetic chromatography (MEKC) (54), liquid chromatography-mass spectrophotometry/mass spectrophotometry (LC-MS/MS) (52), or capillary zone electrophoresis (CE) (53). The LOD obtained by ECL detection is the lowest amongst the above method and is also lower than that obtained from CE-contactless conductivity detection (CE-CD) (14) or LC-MS/MS) (17).

### Real sample analysis

To verify the performance of the sensor in real sample analysis, nisin in egg white or milk was detected using ECL or EC mode. As shown in Supplementary Table S2 (SI). the satisfactory recoveries (94.3–107.3%) and small RSD values (<3.5%) were obtained, indicating good reliability in both ECL or EC detection. This is attributed to the excellent anti-fouling and anti-interference properties of the sensing electrode provided by VMSF nanochannels.

## Conclusions

In summary, we have developed the electrochemical and electrochemiluminescence dual-modality detection of antimicrobial peptides for the first time by combining the interaction mechanism between antimicrobial peptides and cells and the signal amplification effect of VMSF nanochannel arrays. Ru(bpy)_3_^2+^@liposome was easily synthesized and used as both sensing membrane and signal probe. Based on the efficient enrichment of Ru(bpy)_3_^2+^ by VMSF nanochannels, the VMSF/ITO electrode can sensitively detect Ru(bpy)_3_^2+^ probes leaked from nisin-disrupted liposomes in ECL or EC mode, thereby achieving sensitive dual-modality detection of nisin. Combined with the excellent anti-fouling and anti-interference abilities of VMSF, VMSF/ITO electrode can be used for the rapid detection of nisin in milk or egg white sample. The combination of VMSF/ITO sensing electrode and Ru(bpy)_3_^2+^@liposome with integrated sensitive membrane and signal probe provides a new strategy for the detection of non-electroactive antimicrobial peptides. In addition, the high detection sensitivity of ECL and the advantage that EC detection is not affected by the color or transparency of the sample make the constructed dual-modality sensing have an extended sample range.

## Data availability statement

The original contributions presented in the study are included in the article/Supplementary material, further inquiries can be directed to the corresponding author.

## Author contributions

XL: data curation. TZ: data curation and writing—original draft preparation. HT: writing—review and editing. JL: supervision and writing—review and editing. All authors contributed to the article and approved the submitted version.

## Conflict of interest

The authors declare that the research was conducted in the absence of any commercial or financial relationships that could be construed as a potential conflict of interest.

## Publisher's note

All claims expressed in this article are solely those of the authors and do not necessarily represent those of their affiliated organizations, or those of the publisher, the editors and the reviewers. Any product that may be evaluated in this article, or claim that may be made by its manufacturer, is not guaranteed or endorsed by the publisher.
