# Supplementary material for: Novel electrochemical and electrochemiluminescence dual-modality sensing platform for sensitive determination of antimicrobial peptides based on probe encapsulated liposome and nanochannel array electrode
Source: Front Nutr. 2022 Aug 15;9:962736. doi: 10.3389/fnut.2022.962736 (PMC9421287; doi:10.3389/fnut.2022.962736)
Supplement: Supplementary file 1 [file Data_Sheet_1.PDF]

TABLES

Table S1 Comparison between performance for the detection of nisin using different methods.

Detection method	Linear range (μg/mL)	LOD (μg/mL)	Assay time	Ref.
MEKC	2-60	0.3	39 h	54
CE-CD	0.056-9.31	0.02	0.5 h	14
LC-MS/MS	0.1-10	0.04	10 min	17
LC-MS/MS	12.5×10^3-2×10^5	3.4×10^3	12 min	52
CE	10-100	10	40 min	53
ECL	0.01-70	0.0093	10 min	This work
EC	0.8-100	0.07	6 min	This work

MEKC: micellar electrokinetic chromatography; CE: capillary zone electrophoresis; CD: and contactless conductivity detection; LC-MS/MS: liquid chromatography-mass spectrophotometry/mass spectrophotometry.

Table S2 Determination of nisin in egg white or milk.

Detection method	Sample	Added (μg/mL)	Found (μg/mL)	Recovery (%)	RSD (%)
ECL	Egg white[a]	6.40	6.87	107.3	3.5
		10.0	9.43	94.3	2.7
		20.0	20.1	100.5	0.8
EC	Milk[b]	10.0	10.4	104.3	1.7
		15.0	15.3	102.0	3.4
		20.0	14.8	101.2	2.9

[a, b]diluted with PBS (0.01 M, pH=7.4) by a factor of 50.

FIGURES

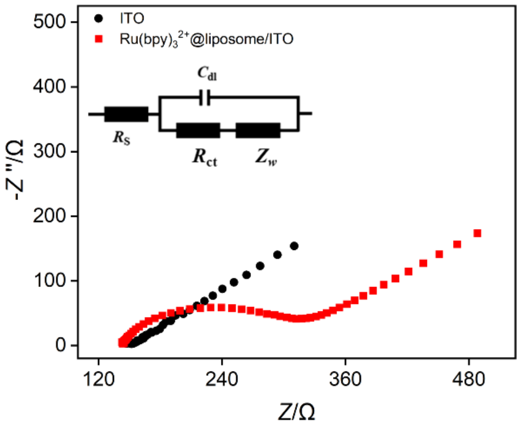

Figure S1 EIS plots of the ITO and $Ru(bpy)_3^{2+}$/ITO electrode in $K_3[Fe(CN)_6]/K_4[Fe(CN)_6]$ (5 mM, 1:1) containing KCl solution (0.1 M). The inset is the equivalent circuit.
